# Characterization of pseudorabies virus transcriptome by Illumina sequencing

**DOI:** 10.1186/s12866-015-0470-0

**Published:** 2015-07-01

**Authors:** Péter Oláh, Dóra Tombácz, Nándor Póka, Zsolt Csabai, István Prazsák, Zsolt Boldogkői

**Affiliations:** Department of Medical Biology, Faculty of Medicine, University of Szeged, Szeged, Hungary

**Keywords:** Alphaherpesvirus, RNA-Seq, Polyadenylation, Gene expression, Viral genomics

## Abstract

**Background:**

Pseudorabies virus is a widely-studied model organism of the *Herpesviridae* family, with a compact genome arrangement of 72 known coding sequences. In order to obtain an up-to-date genetic map of the virus, a combination of RNA-sequencing approaches were applied, as recent advancements in high-throughput sequencing methods have provided a wealth of information on novel RNA species and transcript isoforms, revealing additional layers of transcriptome complexity in several viral species.

**Results:**

The total RNA content and polyadenylation landscape of pseudorabies virus were characterized for the first time at high coverage by Illumina high-throughput sequencing of cDNA samples collected during the lytic infectious cycle. As anticipated, nearly all of the viral genome was transcribed, with the exception of loci in the large internal and terminal repeats, and several small intergenic repetitive sequences. Our findings included a small novel polyadenylated non-coding RNA near an origin of replication, and the single-base resolution mapping of 3′ UTRs across the viral genome. Alternative polyadenylation sites were found in a number of genes and a novel alternative splice site was characterized in the *ep0* gene, while previously known splicing events were confirmed, yielding no alternative splice isoforms. Additionally, we detected the active polyadenylation of transcripts earlier believed to be transcribed as part of polycistronic RNAs.

**Conclusion:**

To the best of our knowledge, the present work has furnished the highest-resolution transcriptome map of an alphaherpesvirus to date, and reveals further complexities of viral gene expression, with the identification of novel transcript boundaries, alternative splicing of the key transactivator EP0, and a highly abundant, novel non-coding RNA near the lytic replication origin. These advances provide a detailed genetic map of PRV for future research.

**Electronic supplementary material:**

The online version of this article (doi:10.1186/s12866-015-0470-0) contains supplementary material, which is available to authorized users.

## Background

Pseudorabies virus (PRV, Suid Herpesvirus 1), also known as Aujeszky’s disease virus, a herpesvirus belonging in the subfamily *Alphaherpesvirinae,* infects swine populations and causes economic losses worldwide. PRV is widely used in studies of the molecular pathomechanism of herpesviruses [1], as a tract-tracing tool for mapping neuronal circuitries [2, 3] and for the delivery of genetically encoded fluorescent activity markers [4]. The transcription of herpesviruses is strictly regulated by cascade-like processes. Three temporal classes of viral genes can be distinguished in terms of the time of their activation during the viral life cycle: initially, the immediate-early (IE) genes are expressed, whose protein products are transcription factors. PRV has a single IE-class gene, *ie180*, which is the major regulator of viral gene expression. The early (E) genes typically play roles in the replication of viral DNA, while most of the late (L) genes code for structural elements of the virus. The PRV genome is arranged into two unique protein coding regions, the unique long (UL) and unique short (US) regions, flanked by the internal and terminal repeats (IR and TR). The genome of PRV is large among viruses, but much smaller than those of cellular organisms, and especially the mammalian genome. The whole transcriptome analysis of PRV can therefore be performed by real-time RT-PCR, a technique, which provides an accurate platform for the temporal analysis of transcription in both wild-type [5] and mutant viral strains [6]. However, PCR can target only a small genomic region, and information related to transcript lengths, splicing, alternative initiation and termination of transcription, unknown transcripts, etc. is not provided. Furthermore, PCR is inconvenient for the detection of novel transcripts. Coding sequences and their related transcripts have been widely studied in PRV [5, 7], together with the microRNA expression in both the lytic and latent phases of the viral life cycle [8, 9], whereas other sources of non-coding transcription, alternative transcript termination and alternative splicing have not yet been analyzed at a genome-wide level. In order to complement previous RT-PCR based studies, we have carried out high-throughput sequencing of the total RNA and polyA(+) RNA fractions of PRV during lytic infection. Transcriptome-wide profiling has led to the discovery of novel regulatory RNAs and an accurate assessment of their expression in several members of the *Herpesviridae* (human cytomegalovirus: [10], anguillid herpesvirus 1: [11]). These studies have discovered highly abundant long non-coding RNAs (lncRNAs), while in addition, the characterization of the MAT ncRNA in murine cytomegalovirus has shown its role not only as a lncRNA, but also coding for an ORF with potential regulatory functions [12]. Host-pathogen interaction studies have also revealed dramatic changes in expression levels of a range of host regulatory- and non-coding RNAs during lytic infection with varicella zoster virus [13]. Recent findings suggest that, similarly as in eukaryotes, alternative transcript termination might be an important regulatory mechanism in herpesvirus gene expression [14]. Indeed, the assessment of 3′ UTRs in PRV strain Kaplan (Ka) identified three genes, each containing two alternative termination sites, while also indicating individual polyadenylation (PA) sites of genes previously recognized as being exclusively transcribed in polycistronic RNAs and not possessing their own PA sites. The PA sites have also been categorized in terms of relative expression levels by determining the overall frequency of proximal and distal PA-site usage per gene.

## Results and discussion

### Assessment of the PRV transcriptome by total RNA sequencing and PA-Seq

For the investigation of the lytic PRV transcriptome, porcine kidney (PK-15) epithelial cells were infected with a high dose (10 pfu) of PRV strain Ka. Samples were gathered up to 24 h post-infection (p.i.) in order to capture all RNA species during lytic infection for sequencing library preparation. Both random hexamer-primed and oligo(dT)-primed libraries were prepared in order to assess total RNA and mRNA transcripts separately. In our modified polyadenylation sequencing (PA-Seq) protocol [14], total RNA was reverse-transcribed by using custom designed oligo(T10-VN) anchored primers containing standard Illumina strand-specific adaptor sequences. The two-nucleotide anchor sequence ensures the annealing of primers at exactly the PA site of mRNAs, providing considerably fewer reads that contain redundant adenine homopolymer stretches, with more useful sequence information resulting for the given depth of sequencing. PA peaks were detected by using HOMER [15] in strand-specific mode, with adjustments for viral cDNA peak calling and a cutoff of 50 reads per base position. PA peaks occurred on both strands, mainly in accordance with previously existing ORF annotations, and also long non-coding RNAs, including the latency-associated transcript (LAT) and the long-latency transcript (LLT).

Both the RNA integrity measurements during the sample preparation, and the low signal-to-noise ratio in the 1 kb region surrounding the PA peaks during the analysis indicated high library quality. Sequencing of the total RNA isolates of infected cells yielded a data set of ~ 208 million 100 bp paired-end reads for the random hexamer-primed library, of which 1.3 million reads aligned to the viral genome version KJ717942.1, and the majority of the remaining sequences aligned to the host organism genome *Sus scrofa* 10.2. PA-Seq resulted in ~ 103 million single- end, 50 bp reads, with 10 million reads aligning to the above-mentioned viral reference.

### PRV transcriptome profiling

**Fig. 1:**
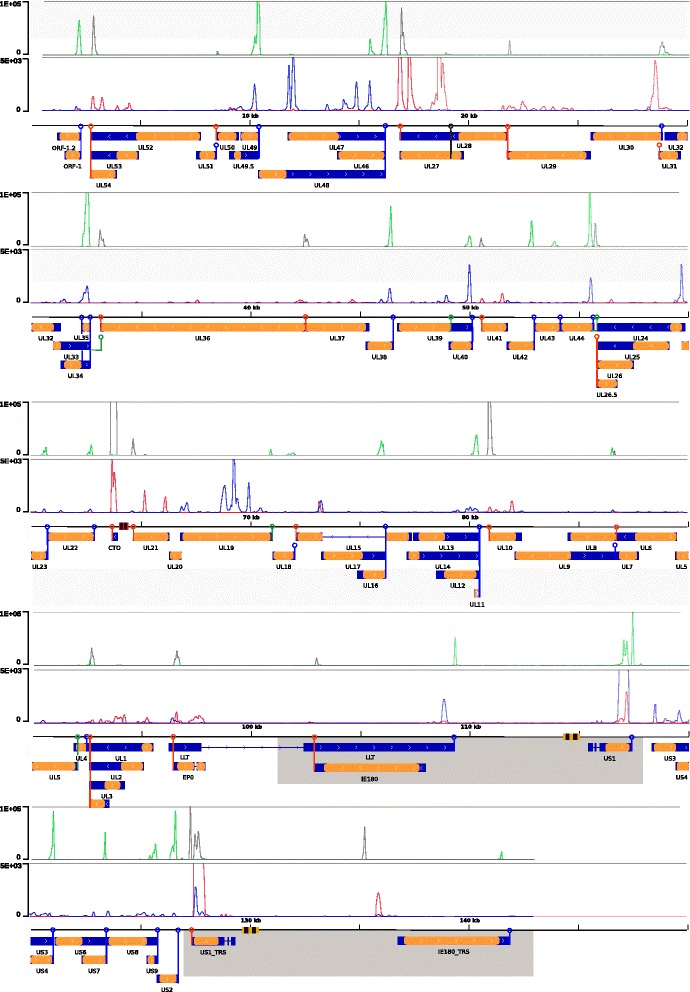
Transcriptional map of the PRV genome identified by total RNA sequencing and PA-Seq. Genetic map: orange: coding sequences, blue: transcripts, red striped rectangle: OriL palindrome, yellow striped rectangles: OriS palindromes, grey: internal and terminal repeat regions, blue circles: PA site on + strand, red circles: PA site on –strand, green circles: alternative PA site on + strand, black circles: alternative PA site on –strand. Expression levels (in coverage per base): upper box: PA-Seq expression, green: +strand read coverage, black: −strand read coverage, lower box: totalRNA sequencing, blue: +strand coverage, red: −strand coverage

Nearly all of the viral genome was transcribed, with the exception of highly repetitive sequences within the terminal and internal repeats that do not encode any RNA species. Similarly, there was no detectable transcription at intergenic repeat regions, which were earlier predicted to be transcriptional insulators [16]. Significant transcription at these insulator sequences was observed only in two convergently oriented gene pairs, *ul44-ul26* and, to a lesser extent, *ul35-ul36*. Here, the alternative transcript termination indicates that leaky transcription traverses the intergenic repeat boundaries with lengths of 109 bp and 443 bp, respectively. On the other hand, non-transcribed, repetitive regions were markedly present between ORF-1 and *ul54*; *ul46* and *ul27*; *ul40* and *ul41*; and *ul11* and *ul10*. In these boundary regions, no expression was observable. A high percentage of the transcription is committed to producing a newly identified non-coding RNA, CTO (“close to OriL”), located between the *ul21* gene and the oriL, between bases 63673–63958 on the complementary strand of genome KJ717942.1. The CTO (RPKM = 1.6×10^6^ in the total RNA library) and US1 (RPKM = 2.32×10^5^) encoding the ICP22 homolog *Rsp40* immediate-early regulatory protein are the most abundant transcripts. Although we examined lytic infection, the two latency-associated transcripts (LAT and LLT) were found to be expressed at a low level, and not at sufficient coverage to determine splicing donor and acceptor sites. Transcription of the hypothetical ORF1.2 [17] sequence was also detected, involving 5′ upstream regions, although single-base localization of the transcription start site is complicated by the presence of several repeats in the genomic sequence in the interval 730–960 bp. On the use of PA-Seq, 3′ transcript boundaries can be accurately identified between and within gene clusters (Fig. 1). In convergently oriented clusters, more extensive overlaps include coding regions of the opposite genes, potentially giving rise to transcriptional interference between the interacting partners [18]. An example of such a relation is between *ul30* and *ul31*, with a tail-to-tail overlap of 80 nucleotides. Here, the expression of *ul30* mRNA exceeds that of *ul31*, with considerable antisense expression over the latter gene, possibly due to transcriptional read-through from *ul30*. As anticipated, convergent genes with more than ~45 bp separating their respective PA signals (PAS) do not demonstrate detectable transcriptional overlap, ranging from *ul18-ul15* (45 bp) to *ul46-ul27* (632 bp), while convergent gene pairs in closer proximity exhibit longer 3′ UTR overlapping regions. A short, 3′-overlapping antisense non-coding transcript (termed SANC) was also found adjacent to the PA site of the *ul21* gene, near OriL (64558–64674 on reference genome KJ717942.1), with an expression of RPKM = 1.67×10^3^ in the total RNA library, the highest non-coding antisense expression in our samples. The various overlaps between the viral genes are presented in Table 1. These overlaps may affect the expression of adjacent genes. It is hypothesized that these interactions form a regulatory network controlling the transcription cascade of herpesviruses [18].

**Table 1:** The organization of alternative splicing, overlapping gene clusters, polycistronic RNAs and alternative polyadenylation events in the PRV genome

Detected splice sites			Alternative polyadenylation			Convergently overlapping gene clusters		Divergently overlapping genes		Tandem gene clusters
Gene	Donor site	Acceptor site	Gene	Alternative polyadenylation signal	Coordinate					
UL15	D −76165	A −73285	UL35	AAUAAA	33133–33138	UL51	UL50	UL52	UL51	UL52-UL54
US1	D +115592	A +115713	UL44	AAUAAA	55768–55773	UL30	UL31, UL32	UL50	UL49.5	UL48-UL46
	D +115766	A +115921	UL22	N/A^a^	63624	UL33, UL34, UL35	UL36	UL29	UL30	UL31-UL32
US1	D −129158	A −129037	UL19	AUAUAAA	71005–710011	UL44	UL26.5, UL26, UL25, UL24	UL32	UL33	UL33-UL35
	D −128984	A −128829	UL28	N/A^a^	18960	UL8, UL9	UL6, UL7	UL37	UL38	UL39-UL40
EP0	D −97480	A −97389	UL5	AAUAAA	92065–92070			UL41	UL42	UL24-UL26.5
	D −97528		CTO	N/A^a^	63538			UL24	UL23	UL17-UL16
								UL21	UL20	UL14-UL11
								UL15	UL14	UL9-UL8
								UL10	UL9	UL7-UL6
								UL6	UL5	UL1-UL3.5
										US3-US4
										US6-US7
										US8-US9

^a^No prediction available for canonical or non-canonical polyA signal using PolyApred server

### Splice sites in the PRV transcriptome

**Fig. 2:**
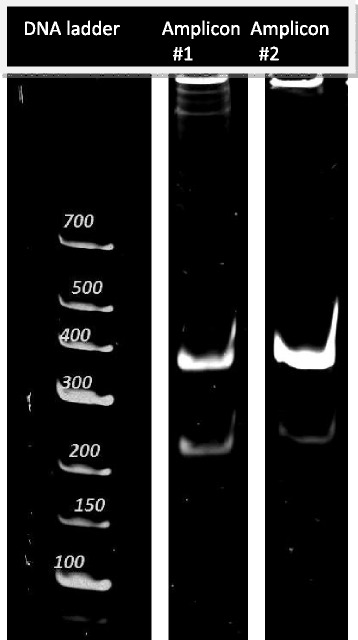
RT-PCR validation of the novel splice site of the EP0 gene, using two different primer sets. 12 % acrylamide gel electrophoresis, Gel Red staining

For splice site analysis, total RNA reads were aligned to PRV strain Ka genome KJ717942.1. All possible splice donor and acceptor sites were considered, with a lower bound of at least 10 supporting reads. Through the exclusion of low-coverage junction candidates, artifacts possibly occurring due to mispriming or template switching during amplification steps could be neglected (Additional file 1). The initial set of splice acceptor and donor sites contained 97 candidate splice junctions, with 49 sites above the threshold coverage. This set contained several permutations of the *us1* 3′ UTR splice junctions, which were screened for the presence of short anchor regions and high mismatch ratios within these anchors. After screening for anchors of <5 bases, consistent splice junctions were readily identifiable. The remaining, high-coverage splice sites are denoted as follows: (D + 10000^A + 12000), with D denoting the donor site, A the acceptor site, and +/− the DNA top and bottom strand, respectively, along the coordinates of the splice junction. Splice sites have previously been characterized in the protein-coding region of *ul15* [19, 20] (D −76165^A −73285), and in the 3′ UTR of *us1* [21] (D +115592^A +115713; D +115766^A +115921), present in both terminal and internal repeats, while one site in the non-coding RNA LLT [22] (D +97765; A +102403) was expressed at an insufficient level for accurate splice site identification. A low percentage of reads also mapped outside the assigned acceptor and donor sites (Fig. 1). A novel alternative splice site was characterized in *ep0*, the homolog of *Herpes simplex* 1 ICP0 [23], which is also a spliced gene, but expressed in the immediate-early class in HSV-1. The newly characterized *ep0* alternative splicing consists of two potential donor sites at (D −97480) and (D −97528) and the acceptor site at (A −97389) (Fig. 1). While the splice junction formed between the acceptor and proximal donor sites conforms to the rule of GT/AG nucleotides comprising ~99 % of junctions in eukaryotic organisms [24], the junction formed with the distal donor site contains GT/CG bases. Experimental validation of the novel splice site has been carried out by RT-PCR, using two primer sets (Additional file 2) designed approximately 100 bp upstream and downstream of the splice site, followed by polyacrylamide gel electrophoresis. The experiments confirmed the presence of the novel isoform during lytic infection robustly after visualization (Fig. 2).

### Frequency of alternative polyadenylation correlated to weak and strong PA signals and flanking motifs

**Fig. 3:**
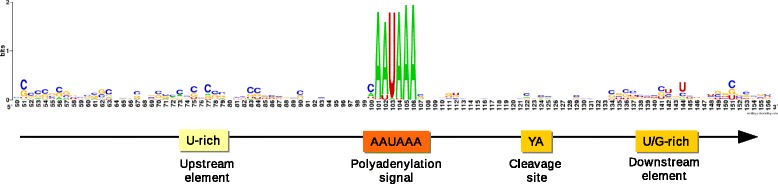
PA signal consensus of PRV and schematic organization of eukaryotic PA sites. The sequence logo represents nucleotide occurrence frequencies within the +/−50 bp region of PA signals in the viral genome. Colored boxes indicate the functional elements affecting eukaryotic polyadenylation

Through the use of the highly sensitive PA-Seq method, the 3′-end of the PRV transcripts was identified by the presence of poly(A) tails. The use of anchored oligo(dT) primers resulted in the accurate mapping of polyadenylation sites, while providing a high coverage for quantitative analyses (Fig. 1). The most highly abundant transcripts (CTO, *us1*, *ul31*, and *ul35*) were in accordance with the random hexamer-primed data, while the greater resolution provided by PA-Seq also allowed the identification of transcripts that were of low abundance or difficult to detect by other sequencing methods or RT-PCR, such as the genes of the *ul6-ul9* convergently oriented cluster. The PAS-usage of eukaryotic organisms is thought to be well conserved, with the canonical AAUAAA being the most widely used signal 10–30 nucleotides upstream of the cleavage site [25, 26]. Not surprisingly, the analysis of the PAS motifs indicated that strong polyadenylation peaks correspond to the AAUAAA signal (<90 %), while AUUAAA is the second most widely used element (~10 %) (Fig. 3). Two further signal motifs were the less conserved USE GU-rich element, >30 nucleotides upstream of the cleavage site [25], and DSE, >20 nucleotides downstream of the cleavage site. In humans, it has been shown that when multiple PA sites are used, the 3′ -most tends to use the AAUAAA signal, while the inner signals tend to vary considerably from the consensus [27]. In our PA-Seq samples, only 6 genomic positions containing the canonical AATAAA sequence proved to be unused PA signals, 3 motifs residing inside coding sequences (+9072-9077; −52929-52934; −78490-78495) and one motif located directly upstream of *us3* (+118308-118313), and not corresponding to any viral transcript. The remaining signal was located within the large repeat regions, and therefore present in two copies, (−117738-117743) and (+126996-127001). On the other hand, canonical PAS that were previously considered inactive demonstrated pronounced polyadenylation peaks, providing alternative transcript termination sites for genes *ul35, ul44* and *ul22*. In both cases, the usage frequency of the distal PA site was at least an order of magnitude lower than those of the proximal ones.

The PA-Seq method additionally revealed polyadenylation peaks in transcripts encoded by upstream genes of tandem gene clusters. These included the polyadenylation of *ul19*. This transcript has previously been detected in strain Indiana-Funkhauser [28], with the non-canonical PAS ATATAAA; in our PA-Seq samples, we have confirmed the active use of this site in strain Ka. A similar arrangement was found in *ul28*, although no conservative PAS was detectable upstream of the well-defined PA-peak at base position 18960. Though PA peaks within the clusters of the US region were markedly above the background signal and correlated well with the coding sequence boundaries, these signals were several orders of magnitude weaker than the commonly observed polyA peaks, making them difficult to validate. The tandemly oriented *ul4* transcript has been hypothesized to be 3′ coterminal with the *ul5* transcript [16], as the canonical PAS directly downstream of *ul5* is inside the *ul4* ORF. However, PA-Seq peaks were found at the 3′ ends of both genes, showing that the PAS of *ul5* is also active. The most abundant transcript during PRV lytic infection proved to be a previously unknown non-coding RNA of 286 bp, located between genes *ul21* and *ul22*, and named CTO. This long non-coding RNA is characterized by an irregular GC composition, where the third-position GC content increases sharply in all three reading frames in the length of the transcript. Based on sequence similarity search, this arrangement is not present in the close relatives of PRV, such as varicella zoster, herpes simplex and bovine herpesviruses. On the other hand, PRV strains Becker, Bartha and HeN1 show <99 % sequence similarity with strain Kaplan in the CTO genomic region. An alternative PA site was also observed, about 120 nucleotides downstream from the main PAS. An in-depth characterization of the transcript is presented in [29].

### Transcription overlaps

**Fig. 4:**
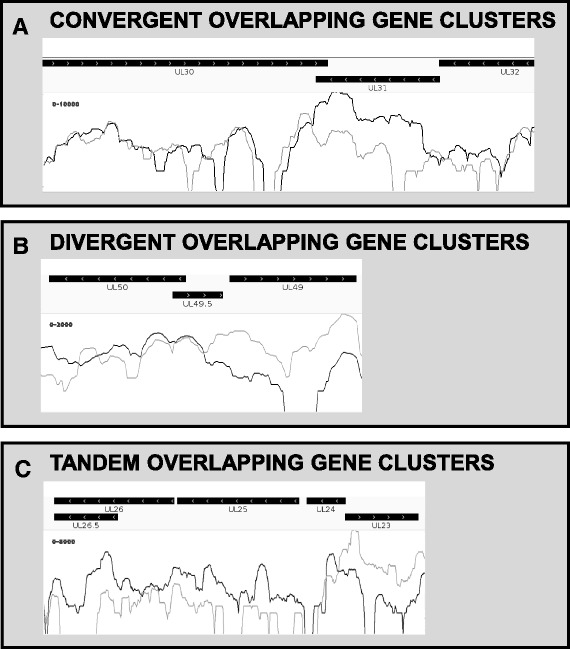
Convergent (**a**), divergent (**b**) and tandem (**c**) overlaps in the PRV genome, as shown by random-hexamer primed samples. Extensive transcriptional overlaps are frequent throughout the condensed viral genome. Black boxes: coding sequences, white arrows: gene orientation, grey line graph: positive strand expression, black line graph: negative strand expression

We assessed the various transcript overlaps, including parallel (tandem), divergent and convergent overlaps (Fig. 4, Table 1). Most of the PRV genes are organized into tandem gene clusters producing polycistronic RNAs (Table 1). An interesting feature of organization is that all of the upstream genes of the clusters end within the downstream genes. Similarly, the divergent gene pairs overlap in every case. Theoretically, this phenomenon may be explained by the restriction of the viral genome length. However, since these overlaps are not too extensive, they probably provide a regulatory mechanism for transcription. The distant convergent genes are separated from each other by repetitive sequences which were found to be heavily methylated (this latter result will be published elsewhere), indicating a mechanism with a likely function for the prevention of transcriptional collisions. Closely located convergent gene pairs transcriptionally overlap or can overlap (alternative transcriptional termination) themselves (Table 1). In the *ul35* and *ul44* genes, these alternative termination sites traversed intergenic repetitive regions, previously considered to be transcriptional barriers. This finding indicates that low-frequency “leaky” transcription occurs more often than anticipated in PRV. Although the function of these PA sites is unknown, it is noteworthy that a highly similar arrangement was present between convergent gene clusters *ul9-ul8* and *ul7-ul6*, with the difference that a strongly repetitive sequence resembling the above-mentioned intergenic repeats in both length and base content, was found within the comparatively long 3′ UTR of *ul7*.

## Conclusion

The single-base resolution map of pseudorabies transcripts revealed that the compact, 143 kbp genome of PRV is transcribed pervasively, with the exception of loci in the large inverted repeats and short intergenic sequences. In addition to previously known splice sites, a novel junction was characterized in the transactivator *ep0*, while the splice sites of lytic genes were confirmed at a high depth of coverage. Polyadenylation signal usage was found to be more frequent than previously predicted, with alternative PAS in genes *ul35*, *ul44*, *ul19*, *ul28* and *ul5*. While alternative transcript termination is a major regulatory factor in eukaryotic organisms, to date there is limited data for viruses in this field. The region of the lytic replication origin was also found to express a novel, highly abundant ncRNA, named CTO, along with a short, 3′ overlapping ncRNA of *ul21*, termed SANC. Other pervasively transcribed regions include the ORF1.2 5′ UTR. The described PRV transcript isoforms and non-coding RNAs help guide future research in the possible regulatory mechanisms of alphaherpesviruses.

## Methods

### Virus, cells and infection

For the propagation of strain Kaplan of PRV, immortalized PK-15 epithelial cells were applied. PK-15 cells were cultivated in Dulbecco’s modified Eagle medium supplemented with 5 % fetal bovine serum (Gibco Invitrogen) with 80 μg gentamycin/ml at 37 °C, under 5 % CO_2_. The virus stock used for the experiments was prepared as follows: rapidly-growing semi-confluent PK-15 cells were infected at a multiplicity of infection of 0.1 plaque-forming unit (pfu)/cell and were incubated until a complete cytopathic effect was observed. The infected cells were frozen and thawed three times, followed by low-speed centrifugation (10,000 g) for 20 min. The cell debris was removed, while the supernatant was concentrated and further purified by ultracentrifugation through a 30 % sugar cushion at 24,000 rpm for 1 h, using a Sorvall AH-628 rotor. The number of cells in a culture flask was 5 × 10^6^. A high multiplicity of infection (10 pfu/cell) was used for the infection of PK-15 cells. Infected cells were incubated for 1 h, followed by removal of the virus suspension and washing with phosphate-buffered saline (PBS). After the addition of new medium to the cells, they were incubated for 1, 2, 4, 6, 8, 10, 12, 14, 16, 18, 20, 22 or 24 h p.i. Mock-infected cells, but otherwise treated in the same way as the infected cells, were used as controls.

### Isolation of RNAs

RNA was extracted from samples of each individual time point of infection by using the NucleoSpin RNA II Kit (Macherey-Nagel GmbH and Co. KG), as described previously [5]. Briefly, after the cells had been collected by centrifugation and lysed with buffer containing chaotropic ions, the nucleic acids were docked to a silica column. The DNA was removed with RNase-free DNase solution (supplied with the NucleoSpin RNA II Kit). Finally, the RNAs were eluted from the column in RNase-free water (supplied with the kit). To eliminate the residual DNA contamination, all RNA samples were treated by an additional digestion with Turbo DNase (Ambion Inc.). The concentrations of the RNA samples were measured by spectrophotometric analysis with a BioPhotometer Plus instrument (Eppendorf). RNA samples were stored at −80 °C until further use.

### cDNA library preparation

Strand-specific total RNA libraries were prepared for paired-end 100 bp sequencing by using the Illumina compatible ScriptSeq v2 RNA-Seq Library Preparation Kit (Epicenter). For polyA-sequencing, a single-end library was constructed through the use of custom anchored adaptor-primer oligonucleotides with an oligo(VN)T_20_ primer sequence. Anchored primers compensate for the loss in throughput due to the high fraction of reads containing solely adenine bases on the use of conventional oligo(dT) primers.

### Illumina sequencing

Transcriptome sequencing was performed on an Illumina HiScanSQ platform at the Genomic Medicine and Bioinformatic Core Facilty of the University of Debrecen. Quality assessment of raw read files was achieved with FastQC v0.10.1. Reads were aligned to the respective host genome (*Sus scrofa*, assembly: Sscrofa10.2) and subsequently to the PRV genome (KJ717942.1), using Tophat v2.09 [30]; ambiguous reads were discarded. For PA-Seq, mapping was carried out with Bowtie v2. [31], followed by peak detection using HOMER in strand-specific mode, with adjustments for the peak qualities of oligo(dT) primed libraries. Peak categories were assigned by using in-house scripts, based on the following criteria: the presence or absence of a PAS in the 50 bp region upstream from the PA site and the presence of at least 2 consecutive adenine mismatches in at least 10 independent reads at the PA site. Annotation and visualization were carried out in the Artemis Genome Browser v15.0.0 [32] and IGV v2.2 [33]. GC bias in the alignments was inspected by using the Bioconductor R package. The prediction of canonical and non-canonical PAS was carried out using PolyApred [34].

### RT-qPCR analysis of alternative splicing

For the validation of splicing events, two sets of primers were designed with lengths from 19 to 23 nucleotides, approximately 100 bp upstream and downstream of the splice site, detailed in Additional file 2. Reverse transcription was performed in 5 μl of solution containing 0.02 μg of total RNA, 2 pmol of the gene-specific primer, 0.25 μl of dNTP mix, 1 μl of 5× First-Strand Buffer, 0.25 μl (50 units/μl) of SuperScript III Reverse Transcriptase (Invitrogen) and 1 U of RNAsin (Applied Biosystems Inc.). The mixture was incubated at 55 °C for 60 min. The reaction was stopped at 70 °C for 15 min. No-RT control reactions (RT reactions without Superscript III enzyme) were run to test the potential viral DNA contamination by conventional PCR. RNA samples with no detectable DNA contamination were used for RT-qPCR reactions.

Real-time quantitative PCR experiments were carried out for each sample in triplicate, on a Rotor-Gene 6000 cycler (Corbett Life Science). Reactions were carried out in 20-μl mixtures containing 7 μl of ×10 dilution cDNA, 10 μl of ABsolute qPCR SYBR Green Mix (Thermo Fisher Scientific), 1.5 μl of forward and 1.5 μl of reverse primers (10 μM each). The running conditions were as follows: [1] 15 min at 95 °C, 30 cycles of 94 °C for 25 s (denaturation), 60 °C for 25 s (annealing), and 72 °C for 6 s (extension). Products were visualized on 12 % polyacrylamide gel stained with Gel Red dye, gel images were acquired using a ProteinSimple AlphaImager HV gel documentation system.

### Availaibility of data

Raw data from PA-Seq and RNA-Seq experiments are deposited in the European Nucleotide Archive under accession code PRJEB9526. The PRV genomic sequence used for mapping is available in Genbank, with accession number KJ717942.1.

## Additional files

Additional file 1:
**Splice site analysis using totalRNA sequencing data.**
Additional file 2:
**Primer sequences for the Real-Time RT PCR analysis.**

## Abbreviations

PRV: Pseudorabies virus
PA-Seq: Polyadenylation sequencing
PAS: Polyadenylation signal
RPKM: Reads per kilobase per million
qRT-PCR: quantitative reverse transcription PCR
