# Supplementary material for: Characterization of pseudorabies virus transcriptome by Illumina sequencing
Source: BMC Microbiol. 2015 Jul 1;15:130. doi: 10.1186/s12866-015-0470-0 (PMC4487798; doi:10.1186/s12866-015-0470-0)
Supplement: Additional file 2: — Primer sequences for the Real-Time RT PCR analysis. [file 12866_2015_470_MOESM2_ESM.doc]

|  | |  | | | |  |
| --- | --- | --- | --- | --- | --- | --- |
| **Amplicon** | **Primer name** | | **Sequence (5'-3')** | **Genomic position** | **Amplicon length (bp)** | **Spliced amplicon length (bp)** |
| # 1 | ep0 splice fw 1 | | tgtcaaacagcgcatcgacgagg | 97230-97252 | 381 | 241 |
|  | ep0 splice rev 2 | | tccaccacgtggacagcga | 97591-97609 |  |  |
| # 2 | ep0 splice fw 3 | | ctggcggttcatcccgtgctc | 97300-97320 | 396 | 256 |
|  | ep0 splice rev 1 | | cacaagttctgtctggactgcatcca | 97669-97695 |  |  |

|  | **Additional file 2. Primer sequences for the Real-Time RT PCR analysis** |
| --- | --- |
